# The Economic and Social Value of Science and Technology Parks. The Case of Tecnocampus

**DOI:** 10.3389/fpsyg.2020.632600

**Published:** 2020-12-23

**Authors:** Jose Torres-Pruñonosa, Josep Maria Raya, Roberto Dopeso-Fernández

**Affiliations:** ^1^Facultad de Empresa y Comunicación, Universidad Internacional de La Rioja, Logroño, Spain; ^2^Escola Superior de Ciències Socials i de l’Empresa, Tecnocampus, Universitat Pompeu Fabra, Barcelona, Spain

**Keywords:** economic value, social value, science and technology parks, input–output model, social accounting matrix, cost benefit analysis, Catalonia, Spain

## Abstract

This article aims to measure both the economic and social value of Tecnocampus, a Science and Technology Park in its region of influence (Mataró city in the Maresme region of Catalonia, Spain). Our results show that the impact of Tecnocampus has a socioeconomic cost–benefit ratio of 2.39. Measuring the impact of this multifaceted centre requires a diverse approach. Although the methods used are not new, the combination of them presents a novel approach to measure the impact of an institution of this nature. We have measured the economic value with the Input–Output model, including the Social Accounting Matrix. On the other hand, for social value calculations, we have used cost–benefit analysis adding measurements of firm localisation to estimate externality effects. Our main results present an economic value of more than 0.054% of the Catalan GDP, whereas the employment impact represents almost 0.37% of total employment in the region. The total economic multiplier of Tecnocampus activity is estimated to be 1.89. Social value generates an additional 0.50 euros to the multiplier according with our analysis. This additional social value represents an increase of productivity estimated in 20 million euros of operational income for Catalan firms and the creation of seven additional firms in the Maresme region as a result of knowledge spillovers. The social value also includes reduction of over-education caused by a better matching between graduates and enterprises, a more direct application of research, and an increase in consumer surplus. Finally, we discuss the policy implications of our findings to promote investments in this kind of infrastructures.

## Introduction

Over the last 30 years, the majority of Europe’s industrial areas, and Catalonia [Nomenclature des Unités Territoriales Statistiques II (NUTS-II)] in particular, have witnessed a transition from an economy with strong links with industry to a service-oriented economy based on innovation. Without question, the weight of industry as a proportion of GDP has diminished conspicuously in many industrial regions, while economic growth depends to an increasing extent on an economy driven by innovation. It is well known that political and economic institutions play a decisive role in the creation of a favourable environment for development, insofar as they establish incentive structures (North, 1990; Williamson, 2000; Acemoglu, 2002). Thus, governments in developed countries strive to promote innovative and entrepreneurial activity (Kuratko, 2005; Baumol and Strom, 2007; Acs et al., 2011; Estrin et al., 2013). In particular, the European Commission regards innovation as a policy that favours quality employment (European Commission, 2009, 2013) in this context. The practical implementation of this strategy has led to the development of numerous public initiatives: incubators, accelerators, investor networks, active networking spaces, technology transfer centres, spin-off facilities, and Science Technology Parks (STPs). STPs (Hobbs et al., 2017) are initiatives commonly implemented by public authorities or universities to create a space for activities based on innovation, under optimal conditions in terms of premises and facilities for R&D, knowledge transfer, and entrepreneurship. The aim of these initiatives is to aid in the transition of so-called industrial districts to so-called innovation clusters. Indeed, the combination of a high degree of industrial clustering, typical of traditionally industrial areas, with a hub based on a high degree of innovation appears to be a factor that facilitates the transition towards an economy based on innovation (APTE, 2007).

Among these initiatives designed to promote innovative activities, we would highlight the promotion and development of a specific STP in Spain. This is the case with Tecnocampus Mataró-Maresme Science Park (hereinafter, Tecnocampus), a non-profit foundation linked to the public university system by an affiliation agreement, a special type of relationship that grants autonomy to create a unique project under the tutelage of the university system.

In this regard, Tecnocampus is affiliated to Pompeu Fabra University, one of the most prestigious universities in Spain. As a matter of fact, Pompeu Fabra University is ranked as the first Spanish university according to the Times Higher Education World University Ranking (2020), the Times Higher Education Young University Ranking (2020), the Ranking, 2019 of research in Sp*anish public Universities* (Buela-Casal et al., 2019), and *U-Ranking* (Pérez et al., 2019). It is ranked as the second Spanish university according to the U-Multirank (2019-2020) and the third one according to the *U.S. News & World Report* (Clarivate, 2019) and the Ranking CYD (2019). Finally, according to CWTS Leiden Ranking (2019), it occupies also the second or the third position, depending on the items measured.

Companies have been mainly considered as generators of economic value, which has placed their social value into a second role (Groth et al., 1996; Retolaza et al., 2015). Nonetheless, some scholars joined both values into a more integrated perspective (Nelson and Winter, 1982; Williamson and Winter, 1993; Torres-Pruñonosa et al., 2012; San-José et al., 2012, 2014; Retolaza et al., 2018). In fact, some methodologies have emerged over the last years to quantify social value of organisations (Lingane and Olsen, 2004; Olsen and Galimidi, 2008; Tuan, 2008; Mulgan, 2010; Retolaza et al., 2016, 2020; Lazcano et al., 2019).

As far as STPs are concerned, most articles deal with their economic impact without measuring their social value creation (Lyra and Almeyda, 2018; Albahari et al., 2019). Fulgencio (2017) establishes the conceptual framework for social value of Bioscience Parks. Lecluyse et al. (2019) critically review both the methods and the theoretical deficiencies of STPs contribution using the Input–Mediator–Outcome framework and suggest future research upon social and human capital theory. Blázquez et al. (2020) propose the use of social accounting to assess the social value of STPs. In this regard, secondary data available in open databases are used instead of primary data.

With regard to higher education industry, most literature focuses on the assessment of the economic value generated by universities (Bonner, 1968; Brownrigg, 1973; Boot and Jarret, 1976; Bramwell and Wolfe, 2008). Drucker and Goldstein (2007) review four approaches taken when assessing the economic regional impacts of universities, namely, “impact studies of individual universities, surveys, production–function estimations, and cross-sectional or quasi-experimental designs” and conclude that their economic impact on the region is considerable. For instance, Garrido-Yserte and Gallo-Rivera (2010) assess the economic value of a Spanish university, University of Alcalá. In actual fact, few are the articles that assess the social value created for society, and most articles deal with the theoretical framework (Vila, 2000; DeClou, 2014). In this regard, Ayuso et al. (2020) quantify the social value generated by Pompeu Fabra University by means of an integrated social value analysis, considering both the economic and the social value that has been created by this university for its stakeholders, using the polyhedral model based on the social accounting methodology (Retolaza et al., 2016). Albahari et al. (2017) analyse the involvement of universities in STPs and conclude that higher involvement is positively related to the number of patents, but negatively related to tenant’s innovation sales.

The aim of this study is to design and estimate the economic and social value of Tecnocampus, an STP, in its region of influence, mainly the city of Mataró (NUTS-V) and Maresme (NUTS-IV) county or region. In methodological terms, the European Commission (2014) guidelines for the economic assessment of investment projects will be followed, as well as the notes for the assessment of STPs (Florio et al., 2008, 2016). Likewise, similar analysis carried out in other STPs and universities will be reviewed, such as Arizona Tech Park (Pavlakovich-Kochi and Vp Research Consulting, 2015), University of Wisconsin-Madison (North Star Consulting Group, 2015), Princeton University (Appleseed, 2016), and other research infrastructures (Raya and García-Montalvo, 2016).

The analysis of the economic value of Tecnocampus’ activity includes the direct economic benefits and indirect and induced effects, which are computed using the Input–Output (IO) model with a Social Accounting Matrix (SAM) for the indirect and induced economic effects. Not only economic sheets, but also surveys administered to entrepreneurs and students are used to calculate the direct economic impact. The calculation of the social value uses the cost–benefit analyses (CBAs) and takes into account a multidimensional perspective, considering the benefits for companies, researchers, students, and the region. We take into account externalities, not only for benefits, but also for costs.

This article is structured as follows: section “Context of the Analysis: The Case of Tecnocampus” will analyse the antecedents and the activities of Tecnocampus. Section “Methodology” will consider the methodology used. The fourth and fifth sections are the “Results” and “Conclusion,” respectively, of the article.

## Context of the Analysis: The Case of Tecnocampus

Tecnocampus is a non-profit foundation created in 2010 that joins together a university campus, an industrial high-tech cluster and a start-up incubator system. The Tecnocampus foundation is promoted by the local government to function as a public–private innovation centre with the objective of fostering the economic and social value of all the implicated agents creating a spillover effect over the region. Therefore, Tecnocampus has a triple-helix formed by local government, industry, and university (Etzkowitz and Zhou, 2018).

Tecnocampus is linked to the public university system by an affiliation agreement, a special type of relationship that grants autonomy to create a unique project under the tutelage of the University system (in Tecnocampus’ case, it is affiliated to Pompeu Fabra University). As a foundation, it is governed by a board in which social and economic actors of the region are represented. These regional ties are essential to view Tecnocampus as a driver of growth through higher education, innovation, and entrepreneurship. It is a regional initiative with a local character within an area with a strong industrial heritage – the city of Mataró and the Maresme region, on the north coast of Barcelona – that has been hit hard by the various economic crises of recent years (Raya et al., 2017; San-José et al., 2020). Mataró is a city where actually industry represents 19% of the GDP, and while standing out in Catalonia as a whole as an industrial area, nowadays it no longer appears to be the great industrial city it was in the 1970s and 1980s when industry represented 50% of the Catalan GDP. In the 19th century, Mataró city and Maresme region were one of the most important industrial cities and regions in Spain and hosted the location of the first railway on the Iberian Peninsula, and the first steam-powered textile factories. During the 20th century, the industrial sector not only has expanded, but also has diversified from textiles into other related industries: industrial machinery, metallurgy and chemicals, and, many years later, information technology. Since its inception, the project was led from within the region, with the influence of the City Council (historically closely involved in education, Mataró being one of the cities in the country in which the City Council’s influence in education is most marked) and the backing of businesses, which became partners in an educational model always focussed on preparation for professional practice and grounded in the importance of work placements in their production plants. Business organisations were represented in its governing body from the outset and had an influence on study programmes and on a dual educational system in which training in entrepreneurship was considered a core subject in study programmes for future engineers.

To explain the impact of Tecnocampus on the region, it is relevant to take into account these antecedents of entrepreneurship and early industrialisation in the region, which over the years gave rise to a culture of entrepreneurship, which is a special characteristic of the local society. There is no doubt that to explain Tecnocampus’ success, it is necessary to understand its historical links with the local manufacturing industry and to the Catalan entrepreneurial culture and the Catalan government, which has its own heritage and identity within the Spanish State. This culture of industry and entrepreneurship permeates society, acting as an informal institution. In other words, it creates a set of informal rules, ways of acting, and perceiving that come together to forge a natural attitude towards the decisions involved in creating individual initiatives (Audretsch, 2007).

The Tecnocampus project involved the development of a built area of 50,000 m^2^ and a total investment of 50 million euros, and today, 10 years after it began operating, it can be considered a success. The explanation for the success of the model may be attributed to the interaction between business and their surroundings, a true ecosystem in which university and business go hand-in-hand. This success has been quantified in this study on the basis of the initiative’s impact on the area, which is evident in the growth data presented.

Tecnocampus has three university centres: School of Business and Social Science, School of Engineering and Technology, and School of Health Sciences with 293 professional teachers. There were 3,535 students who have been enrolled from 2010 to 2019, in 12 official undergraduate and 11 graduate degrees. Ninety-one percent students chose Tecnocampus as the first option when selecting their university to enrol. Tecnocampus has bilateral agreements with 86 universities in the Erasmus framework, 25 universities in the rest of the world, and 802 company collaboration agreements. The incubator has provided a location for 21 start-ups, and the consultancy service for business creation has created 60 companies in 2019 and has provided support to 524 entrepreneurs. Currently, the STP is complete, within the region of 120 innovation-based businesses that employ approximately 823 employees, and with an enlargement of the complex underway to accommodate a level of growth that has surpassed the initial expectations.

All in all, Tecnocampus is an institution whose mission is focussed on the social and economic development of the region (Maresme, Barcelona north coast) based on two approaches that are organised within a holistic model that aims to blend them into a whole: (1) higher education strongly oriented towards preparation for professional practice and (2) the activities of an STP that include the accommodation of innovative businesses and the incubation of start-ups.

## Methodology

As we have already mentioned, Tecnocampus is the combination of three university centres affiliated to the Pompeu Fabra University and a Technology Park, managed jointly in order to achieve an actual virtuous circle between university and business. STPs, besides fostering the regional economic development, contribute to the improvement of the region’s quality of life by improving its human capital, creating jobs with an added value as well as research an innovation. These three aspects are highly related to long-term growth in a region.

All these activities have significant effects on the geographic area under the STP’s influence in terms of economic development, both with regard to the generation of economic activity and in the creation of employments. Not only the expenses necessary for the STP’s functioning must be taken into account, but also the expenses of the STP’s firms and the students coming from outside the area of influence, all of which result in an injection of revenue and the generation of local production and occupation.

Measuring the impact of this multifaceted centre requires a diverse approach. For this reason, this article combines different methodologies to measure the economic impact of this institution. Despite the fact that the methods used are not new, the combination of them presents a wider picture of the impact of an institution of this nature.

In economic literature (Fletcher, 1989; Meng et al., 2013; Chang et al., 2014; Stadler et al., 2014; Artal-Tur et al., 2016), the tool most commonly used to estimate the economic value or profit of an infrastructure is the study of the economic impact by means of the IO charts, which allows measuring the effect of the interdependence among the different production sectors, distinguishing between direct, indirect, and induced impact.

The attractiveness of the economic impact studies based on the use of multipliers by means of IO charts is their limited needs of information, in comparison to the demands in terms of modelling and information of other methodologies.

Nevertheless, some studies (Lee and Taylor, 2005; Bonfiglio and Chelli, 2008; Flegg and Tohmo, 2013; Gretton, 2013) defend that in these works on economic impact, net profits are overestimated, and they suggest that other economic assessment techniques, such as the CBA, should be used in the economic and social assessment of these events.

The studies on economic impact and CBA (Brent, 2006; Mishan and Quah, 2007; Raya-Vìlchez and Moreno-Torres, 2013; Sartori et al., 2014; Boardman et al., 2017) are effective to convey to the society, in a quantified way, the economic and social effect of a particular infrastructure or public policy. To such an extent that when dealing with supranational entities financing jointly an investment, this kind of studies is usually required for them to be discussed and potentially approved. Thus, studies on the economic impact of big science infrastructures and their updating, either periodical or with the current data, instead of previsions, are usual economic exercises. Many a time, the updating of the original studies refines models and allows working with actually made investment instead of with previsions.

The methodology used to assess the overall impact (both economic and social) of Tecnocampus is based on different types of analyses. We have measured the *economic value* based on the IO model, including the SAM. On the other hand, as far as the *social value* is concerned, two methodologies are used. First, a CBA to capture all the possible non-economic costs and social returns from Tecnocampus activity has been used. Second, a regional analysis based on different measurements of firm localisation to estimate the externality effect of Tecnocampus in its region and the spillover effects has been applied.

The data for the analysis were obtained from internal records of the institution, surveys to other stakeholders, and SABI^1^ database. The financial records of Tecnocampus were used to value the direct expenditure of the institution. Surveys were applied to firms and students to capture other economic activity inside of the STP that is not assessed by the financial reports of Tecnocampus. SABI was used to monitor the creation and impact of new firms in the region since the inception of Tecnocampus so as to assess the external effects.

Economic value was divided into three main categories: (1) direct impact, (2) indirect economic impact, and (3) induced effects.

First, the direct economic impact of the Tecnocampus has been split into four categories: operating expenses, investments, start-up expenses, and expenses of visiting students.

Second, for the IO analysis, an IO matrix of the economy and relationship between economic sectors is needed to estimate the indirect economic impact of expenses. Therefore, the latest Catalan IO matrix available published by the Catalan statistical office (Idescat), was used. Additionally, an aggregated expenditure for Tecnocampus was needed.

Third, induced effects are estimated, and in this regard, macroeconomic accounts are necessary. The link between the R&D industry and macroeconomy is obtained by inserting the Catalan IO matrix into a SAM, which presents a snapshot of the economy for a given year by means of a double-entry table that synthesises and describes the structure of an economy in terms of the links between production, income distribution, and demand. Thus, the revenue and expenditure of all agents and institutions in an economy are included. As a square matrix that records flow of all transactions (by equalising total expenditures/leakages to total incomes/injections), it provides a balanced macroeconomic position.

Social value was divided into three main categories: (1) for companies, (2) for researchers and students, and (3) for the region.

First, the social value created for companies can be assessed by the number of spin-offs and start-ups, the development of new products and processes, and the provision of special services and knowledge spillovers to non-user businesses, which prefer to be located close to the STP. Both the social value of the jobs created and the R&D investment were monetised by means of CBA.

Second, social value created for researchers and students considers four items, and also CBA was used: (I) the value of scientific publications. In this regard, both the Tecnocampus researchers’ wages and the time they spent in research were used. (II) The reduction of over-education for both researchers and students. (III) Social value for students is captured by the reduction of the future rate of unemployment and by a higher future salary (approximately 7.15%, according to de La Fuente and Jimeno, 2011). However, this benefit cannot be attributed to all students but only to those who without the Tecnocampus would not have continued their studies. (IV) The consumer surplus for some students has been monetised.

Third, the social benefits for the region have been calculated on the basis of the seminal work by Rosenthal and Strange (2003), which states that the firm’s location decision is based on the maximisation of profits. The local characteristics displace the production function, so firms will decide to be located in regions that foster their productivity. Based on this model, we conducted a regression analysis using as dependent variables the number of new firms in the region and, as a second step, an ordinary least squares (OLS) of the profit generated by the firms in the region to capture also external effect to the existing firms. Using data before and after the creation of Tecnocampus, we can measure the external effect on the location of start-ups (number of new firms) and productivity (profits) of firms in different regional setups.

### Economic Impact of the Tecnocampus STP: Direct Impact and Input–Output and SAM Analysis

This subheading deals with the analysis of the economic impact originating by Tecnocampus activity. The amount of the money spent by the STP, students, or start-ups in their daily activity generates economic impact. Besides the direct impact, indirect and induced impacts are calculated. To assess both indirect and induced effects, the IO model, including the SAM, is the most used methodology. As a matter of fact, IO models take into account interindustry IO relations and final demand (i.e., exports and imports, investment, consumption, and so forth) simultaneously (Pyatt and Round, 1979). Thus, the impact of an external demand shock on the economy (for instance expenses from start-ups or students) can be estimated. The Tecnocampus activity demands services from several sectors or subindustries, and therefore, any demand or/and supply-side shock given to the industry involves industrial and interindustry impacts. The main input used to calculate the economic impact by means of the IO analysis is the current spending by students and start-ups, which is usually determined by means of surveys. Furthermore, the economic sheet of the foundation is important to calculate the impact of operating expenses and investments. The multiplier effect that the spending of one category has on the host economy is a usual result. Therefore, the multiplier effect is the increase in final income due to knock-on effects within the local economy from new extra spending in this category. In actual fact, it comprises the direct, indirect, and induced multipliers. First, direct effects are the increase in sales revenues of firms’ or students’ spending. Firms, in turn, need to purchase inputs from other firms located in the region, which, in turn, will have to purchase inputs from other ones and so forth. These are the indirect effects we refer to, which are generally distributed among various economic sectors, in contrast with only those that are most directly associated with Tecnocampus’ activity, which is the case of direct effects from firms’ expenses. Finally, induced effects are generated when the receipts (increased incomes, such as employees’ wages) of direct and indirect expenditure are spent. This generates further consumption, input spending by firms, and so on, ultimately generating an increase in output, added value, and employment in the host economy. Hence, the final increase in income in the host economy is generally higher than the initial increase generated by firms and students’ spending. SAM models that include IO models are commonly used to obtain income multipliers of induced effects. The spatial dimension of these effects has restricted the area to Catalonia.

Despite the fact that the limitations of the traditional IO economic impact model are well known (Czamanski and Malizia, 1969), it is still commonly used in many sectors. Criticisms of traditional IO models include the fact that relative prices are fixed, and consequently, input substitution is not possible, factor inputs are infinitely available, and there is a linear relationship between direct and indirect effects. Because of all these limitations, a positive increase in the demand of a region will always cause an expansion of its economic activity, as well as positive multiplier effects. Hence, economic impacts may be overestimated. Nonetheless, other kinds of models (such as general equilibrium models) require a large amount of information that, as in our case, is not always available. Consequently, we have employed a traditional IO model to analyse the economic impact (direct plus indirect effects) of investing in STPs and a SAM for calculating induced effects.

The total multiplier from previous studies on STPs and higher education institutions (Moretti and Thulin, 2013; Hermannsson et al., 2014; Zhang et al., 2017) generally ranges between 1.51 and 2.03. Therefore, it is expected that the multiplier obtained in our case will be within that range.

### Questionnaire Data

In order to estimate the direct economic impact, a survey was carried out to all students from outside of Catalonia and all start-ups, during May 2017. With regard to students, 118 students came every year from outside of Catalonia to take their undergraduate and graduate courses (93 are undergraduate and 25 graduate). We collect 56 valid responses from an online survey. Therefore, using a 95% of confidence, the margin of error was 5.35%. The questionnaire mainly included questions regarding expenses and their composition (accommodation, shopping, transport, etc.). The expenses of the Tecnocampus students from outside of Catalonia in 2016 were almost 0.9 million euros. The main recipient was the trade, transportation, and hospitality sector.

As mentioned before, 120 start-ups are set in the Tecnocampus STP. Through a questionnaire, each company was asked about the number of employees, salaries, investment in R&D, number of patents requested and granted, and their main financial figures (turnover, expenses, etc.). Finally, 59 valid responses were obtained, and the margin of error was 5.8%. The expenses of the start-ups in 2016 amounted to more than 61 million euros. The main recipient was the industry sector.

### Cost–Benefit Analysis

In a CBA, economic benefits and other social benefits of investing in STPs, as well as economic and non-economic costs, are taken into account. Therefore, this methodology makes it possible going one step further and evaluating some social variables and effects that are not taken into account by other methodologies such as IO outcomes.

The costs and benefits included in a CBA are social in nature (i.e., social costs and social benefits) and included monetary and non-monetary, as well as tangible and intangible, costs and benefits. CBA measures all benefits and costs in monetary terms, so that a single measure of “social profitability,” the “net benefit” (net present social value), can be obtained. If this figure is positive, net benefits are positive; i.e., social benefits exceed costs, and hence the investment is socially profitable. This way of proceeding also allows the comparison of alternative uses of resources or funds and therefore allows the decision-maker to make investment decisions by comparing the net social value of alternative investments. CBA is commonly used in public sector investment decisions; in fact, in many cases, public projects have to pass a CBA before being implemented, to show and quantify their net social value.

In order to obtain a single figure of net social value, CBA needs to value all costs and benefits (present time equivalent) in monetary terms, including those that do not have a market price (intangibles; Florio et al., 2008). The estimation of the economic benefits obtained from students’ spending and the economic impacts in the previous section was easier because things such as board and lodging or ports equipment, to mention just a few examples, have market prices. The valuation given by individuals to goods or services as a measure of their willingness to pay for them is captured by these market prices under some assumptions (such as perfectly competitive markets). With regard to intangibles, there is no market price, and consequently, there is no observable monetary figure for individuals’ valuations. Several methodologies can be used to value intangibles. The basic methods are revealed preference methods (indirect methods) and stated preference methods, such as contingent valuation methods (direct methods). The revealed preference methods are based on an individual’s market decisions (individuals paying or accepting compensation by buying or selling, for example), which can be used to “reveal” how individuals value the intangible. Among the most commonly used methods, it is worth mentioning the travel cost method, hedonic prices, human capital models, and productivity models. Below, a first approach to a CBA of public investment in STPs is presented.

### Regional Impact Analysis

The objective of the location exercise is to evaluate how local conditions affect the decision of new companies to locate within the territory. Therefore, starting from the classical theoretical basis, a variable that measures the presence of Tecnocampus was added. The aim is to quantify the effect within the territory and compare the main differences in order to draw conclusions about it.

The theoretical model is based on the work of Rosenthal and Strange (2003). It is based on the fact that each firm will make its own decision on location through a profit maximisation condition where the price of production is normalised to 1.

(1) π⁢(y)=a⁢(y)⁢f⁢(x)-c⁢(x)

where *a*(*y*) displaces the production function *f*(*x*), *y* is a vector of local characteristics, and *x* is a vector of inputs that have a cost *c*(*x*). The inputs are chosen to maximise benefits in the traditional way. Thus, for example, the company maximises on the optimal number of inputs *x* in such a way that:

(2) a⁢(y)⁢d⁢f⁢(x)/d⁢x-d⁢c⁢(x)/d⁢x= 0

The birth of an establishment occurs if it can obtain positive benefits when its inputs are at maximisation levels. At the same time, as businesses are heterogeneous in their potential to generate profits, Equation (1) can be rewritten as follows:

(3) $$\pi \,\left(y,\varepsilon \right)=\max_{x}a\,\left(y\right)\,f\,\left(x\right)\,\left(1+\varepsilon \right)-\,c\,\left(x\right)$$

where *ε* is independently and identically distributed through all possible locations according to a cumulative probability function Φ (*ε*). In this way, for each value of *y*, there is a *ε ^∗^ (y*) for which π (*y, ε ^∗^* (*y*)) = 0, so for a π (*y, ε*) > (<) *0*, there is a value of *ε* (*y*) > (<) *ε ^∗^* (*y*). Therefore, the probability that an establishment is created will be equal to Φ (*ε ^∗^* (*y*)). Assuming that the presence of Tecnocampus in the region is a local characteristic found in the vector, it would be expected to increase the probability that a business would be established in the region.

Although this model was developed to be the basis for the theory of location in urban areas to account the advantages of agglomeration economies, it can be assumed that the Maresme region can be accounted as an urban entity formed by several municipalities with Mataró as the urban centroid.

To capture empirically these regional effects, panel data with fixed effects regression model have been used. The dependent variable, the number of new firms, is explained by a series of local characteristics of the municipality. Therefore, the model to be estimated would have the following form:

(4) $$N_{i\,t}=\beta^{1}\,N_{i\,t-1}+\beta^{2}\,T\,C\,M_{i\,t}+\beta \,X_{i\,t}+\eta_{i}+\varphi_{t}+\varepsilon_{i\,t}$$

where *N*_*it*_ is the number of new firms in municipality *i* and in period *t*, that is, the dependent variable that we seek to predict. The independent variables are made up of a lagged value of new firms *N*_*it–1*_ in order to measure the persistence of births and capture how new companies follow other entrepreneurs also known as location economies. The variable *TCM*_*it*_ is the one used to measure the presence of Tecnocampus by means of a dummy variable. The matrix includes another series of regressors that measure local conditions *X*_*it*_, such as population, municipal public spending, presence of credit institutions, and population density, among other relevant variables. The variables η_*i*_ and φ_*t*_ are municipal and temporal fixed effects, which try to capture the unobserved heterogeneity resulting from other factors that are common to a territory (municipality) or to a specific year within the study period. Finally, ε_*it*_ is the error term that captures the part of the dependent variable that cannot be explained by the model.

A second version of the model previously presented is calculated in order to account for the productivity effect of Tecnocampus on existing firms.

(5) $$I\,n\,c\,o\,m\,e_{i\,t}=\beta^{1}\,I\,n\,c\,o\,m\,e_{i\,t-1}+\beta^{2}\,T\,C\,M_{i\,t}+\beta \,X_{i\,t}+\eta_{i}+\varphi_{t}+\varepsilon_{i\,t}$$

The variable *Income*_*it*_ captures the total operation income of the firms located in Maresme, so we can account for additional productivity not only in the new firms created but also the spillovers in existing firms.

To estimate the model, data from the period 2002–2011 have been used, as more current data are not available for some of the relevant variables. This period focussed on the effect of Tecnocampus in its first 2 years of existence, which is expected at least to be maintained over time. The data on new firms and total operation income of existing companies are obtained from SABI database, although it is not a census of companies, it allows to obtain data at the municipal level that is not available in public datasets. The Ministry of Finance database has also been used to approximate local public spending, and the statistical database of the largest Spanish savings banks (La Caixa, 2013) is used to obtain municipal activity data. A total of 280 observations are obtained over 28^2^ municipalities and 10-year period.

## Results

### Economic Value: IO Analysis

We have divided the direct impact of the Tecnocampus into four parts: operating expenses, investment expenses, expenses of the start-ups, and expenses of the students from outside of Catalonia. To carry out the IO analysis, and consequently to calculate the indirect economic impact from the expenses, it is necessary to have an IO table of the Catalan economy, as well as the relationship between economic sectors. We have used the latest Catalan IO table that was available when the analysis was carried out, generated by the Catalan statistical office, Idescat, for 2011. Although we present the information for the grouping of 10 sectors, we have used the grouping of 82 sectors for calculations. An aggregated expenditure for the Tecnocampus was needed.^3^ As stated above, this figure was obtained from the information given in the surveys collected, as well as economic sheet of the company. Thus, indirect effects of the Tecnocampus were calculated using the well-known IO table multipliers (Tables 1–3). Direct plus indirect effects were 96,924,866 euros in terms of output; 33,517,817 euros in terms of added value and 1,349 jobs in terms of employment.

**TABLE 1:** Total impact of Tecnocampus activity.

	**Direct**	**Indirect**	**Induced**	**Total**
Output	78,264,517.46	18,660,348.72	21,601,933.68	118,526,799.86
Value added	24,051,765.97	9,466,050.89	12,293,958.76	45,811,775.62
Employment	950.72	398.68	486.71	1,836.11

**TABLE 2:** Total impact of Tecnocampus activity by sectors. Output in euros.

	**Direct**	**Indirect**	**Induced**	**Total**
Primary sector	0.00	0.00	0.00	0.00
Industry	29,326,487.50	11,624,476.56	13,495,752.14	54,446,716.20
Construction	245,479.07	210,838.42	110,472.14	5,663,789.63
Commerce, transport, and hospitality	2,184,830.97	1,059,934.23	1,137,411.85	4,382,177.05
Information and communication	1,536,306.96	316,757.49	546,845.94	2,399,910.39
Financial activities	1,993,468.31	1,047,874.95	719,734.34	3,761,077.60
Real estate activities	0.00	0.00	0.00	0.00
Professional activities	5,506,375.47	3,016,746.65	2,389,228.85	10,912,350.97
Health, education, and public sector	4,019,695.34	1,252,829.71	3,075,304.05	8,347,829.10
Entertainment activities	344,994.55	130,890.71	127,184.37	603,069.63
Total (without domestic economies)	45,157,638.17	18,660,348.72	21,601,933.68	85,419,920.57
Domestic economies	33,106,879.29			
Total	78,264,517.46			118,526,799.86

**TABLE 3:** Tecnocampus economic multipliers.

	**Type I**	**Type II**
Agriculture	0.00	0.00
Manufacturing	1.40	1.86
Construction	0.00	0.00
Commerce. transport and hospitality	1.49	2.01
Information and communications	1.21	1.56
Finance and insurance	1.53	1.89
Real state	0.00	0.00
Professional activities	1.55	1.98
Public sector, education, and health	1.31	2.08
Culture, entertainment, and others	1.38	1.75
Total	1.41	1.89

The standard IO model is useful in the estimation of the indirect economic impact resulting from Tecnocampus’ expenditure, as it includes interdependence among production industries in an economy and provides information about intermediate and final demand. However, it is not enough to calculate induced effects. Induced effects are created when employees and anyone whose income increases in general spend the new income generated in the region by Tecnocampus’ spending. Consequently, there is an additional effect on final demand. Macroeconomic accounts are required to calculate induced effects. The link between the R&D industry and the macroeconomy is obtained by inserting the Catalan IO table into a SAM. A SAM presents a snapshot of the economy for a given year. It is a double-entry table that synthesises and describes the structure of an economy in terms of the links between production, income distribution, and demand. The revenue and expenditure of all agents and institutions in an economy are included (Thorbecke, 1998). As a square matrix that records flows of all transactions (by equalising total expenditures/leakages to total incomes/injections), it provides a balanced macroeconomic position.

The SAM built by Llop (2012) has been used to calculate induced economic effects. It covers consumption–income relations in the Catalan economy. Table 1 shows that including induced effects in the computations increases the previous economic impact figures, which only included direct and indirect effects. As can be seen in Tables 1, 2, total impact is 118,526,800 euros in terms of output; 45,811,776 euros in terms of added value, and 1,836 jobs in terms of employment. These figures are significant in relative terms. The output impact represents more than 0.054% of the GDP^4^ whereas the employment impact represents almost 0.37% of total unemployment. Finally, the total multiplier generated by Tecnocampus activity is 1.89 (Table 3), in line with those previously found in the literature, which range from 1.51 to 2.03 (Moretti and Thulin, 2013; Hermannsson et al., 2014; Zhang et al., 2017).

### Social Value: CBA of Tecnocampus Activity

One mischievous procedure of economic impact calculation by using IO tables has to do with the fact that it considers only economic impacts and, in consequence, only economic benefits, ignoring the increase of non-monetary costs that can also be generated by Tecnocampus’ activity. A CBA allows the inclusion of these as well as other social costs and benefits. This subsection presents a CBA of public investment in Tecnocampus. In this analysis, the costs carried out by the local community and opportunity costs (benefits if resources were redirected to other activities) are included. Furthermore, the non-monetary benefits that cannot be included in an economic impact analysis are also included. In particular, this research includes benefits for companies, researchers, and students and benefits for the region (Florio et al., 2008; Florio, 2014).

The first aspect to consider when carrying out a CBA is the establishment of a time horizon. The time horizon is particularly relevant for investment projects where the costs and benefits streams are generated in different moments (usually, costs first and benefits later). In our case, the initial investment in Tecnocampus was made from 2003 to 2010. In this respect, we are interested in social net value of Tecnocampus yearly activity rather than the whole expected flows during the investment period. Accordingly, the analysis presented below corresponds to 1 year, 2016, and no net present value calculations are necessary.

A further issue is which benefits and costs should be considered in a CBA. In this article, they have been chosen according to the literature on investment in STPs and higher education (Florio, 2014) and the possibilities of estimating them using available information. The benefits for the population of Catalonia are as follows: Tecnocampus’ economic revenues, benefits for companies (start-ups creation, innovation, and location), and benefits for researchers and students (research, reduction of over-education, consumer surplus, and access to innovation programmes). Finally, although not valued, we will offer some discussion about region benefits. With regard to costs, tangible costs have been included (operating and investment costs of Tecnocampus activity), as well as local students’ costs. In all cases, the spatial dimension considered is the Catalonian area.^5^ The selection criteria for the chosen benefits and costs are data availability and expected value of the figure. An example could help to illustrate this point. Ignored costs could be either negative externalities (in terms of a higher traffic)^6^ or positive externalities (an increase in housing prices). In this case, it is not only difficult to know the exact figure, but also the relative decrease in figures. That is, concepts that we have not taken into account are negligible.

#### Social Benefits

##### Social benefits for companies

Concerning social benefits for companies, these benefits can be in the form of the establishment of spin-offs and start-ups, the development of new products and processes, and the provision of special services and knowledge spillovers to non-user businesses, which prefer to locate close to the STP facilities. With respect to the creation of new businesses, they should be valued as the expected shadow profit gained by the business during its lifetime, as compared to the counterfactual situation (either by a higher survival rate or by the fact that this is a business that had not been created). Because of lack of data to implement this calculation, we use a traditional valuation based on the economic value of the jobs created. Within this framework, we can value the companies of the STP in 3,719,237 euros. This valuation of start-up creation can be regarded as conservative for several reasons. First, the salaries we are using are below the market price due to the high unemployment in the area. Second, given the high innovative profile of start-ups, it can be considered that the value they have in this initial phase is under their average lifetime value.

Benefits can be in the form of new products. Thus, when patents are registered at national, European, or other patent offices, their benefit can be estimated by their economic value. Two patents were registered in 2016 at Tecnocampus, 1 for every 68 researchers.^7^ However, since it has been recently registered, it is difficult to value this figure. Finally, an alternative way of valuing innovation is to use the spending on innovation of the STP’s companies. According to data from the survey conducted to the start-ups, its average investment in R&D has already been 3,601,591 euros; this figure is more than five times its turnover. This is usual if a high percentage of the companies have been recently created. If we assume this figure as a long-term investment made and set a standard maturity period of 20 years, the average annual investment of the companies of the STP would be 180,080 euros per year. This figure represents more than 25% of the current turnover of these companies. Therefore, investment in R&D of all the companies in the STP amounts to 2,160.955 euros. Bornmann (2013) indicates that the social return rate of private R&D is approximately 50%, which would indicate that the economic value of this investment in R&D is 3,241,432 euros; that is, 1,080,488 euros of this value is a benefit.

Finally, benefits to locate close to the STP are valued. External businesses are incentivised to locate in the STP because they have the opportunity of acquiring new knowledge and technological skills spilling over as externalities from the RDI facilities. Additionally, external businesses can benefit from the network of knowledge and contacts that involves locating close to the Tecnocampus. The localisation effects estimated by Eq (5) are presented in Table 4. The first column (M1) presents the effect of local determinants in the operating income of all firms in the region, whereas the second column (M4) adds the Tecnocampus effect on the productivity of firms. We can observe that the presence of the STP in the region has an average positive effect per year of 53.1 million euros on the total operating income for companies in the region of Maresme. The above figure in percentage terms represents 0.85% of the total income of companies in the Maresme region in our sample. This will suggest that the presence of Tecnocampus in the region helps fostering the local productivity in 85 cents for each 100 euros in sales. An analogous exercise can be carried out for the whole of Catalonia. The presence of Tecnocampus in the region has an average positive effect of 20.5 million euros. This figure represents 0.01% of Catalan companies’ total income. Extending the regional scope of the STP reduces the overall impact on existing companies; this suggests that the social value can be better captured in more local settings.

**TABLE 4:** Tecnocampus productivity localisation effects.

	**M (1)**	**M (2)**
	
	**Base (productivity)**	**TCM effect (productivity)**
	**Coef. (s.d.)**	**Coef. (s.d.)**
Dependent variable (*t*-1)	0.30453***	0.30453***
	(0.04873)	(0.04873)
TCM effect		5.31e + 07***
		(1.85e + 07)
Local public expenditure	−0.49441	−0.49441
	(0.73027)	(0.73027)
Population	2.40e + 04***	2.40e + 04***
	(5.00e + 03)	(5.00e + 03)
Unemployment rate	−5.91e + 06*	−5.91e + 06*
	(3.09e + 06)	(3.09e + 06)
Telephones	−2.34e + 04***	−2.34e + 04***
	(5.68e + 03)	(5.68e + 03)
Automobiles	−1.02e + 02	−1.02e + 02
	(1.15e + 04)	(1.15e + 04)
Trucks and cargo vehicles	−1.86e + 04	−1.86e + 04
	(4.77e + 04)	(4.77e + 04)
Other vehicles	−1.77e + 04***	−1.77e + 04***
	(6.03e + 03)	(6.03e + 03)
Banks	−3.89e + 06	−3.89e + 06
	(3.83e + 06)	(3.83e + 06)
Savings banks	3.94e + 06	3.94e + 06
	(2.56e + 06)	(2.56e + 06)
Credit unions	1.61e + 07	1.61e + 07
	(2.64e + 07)	(2.64e + 07)
Retail commerce	1.18e + 05***	1.18e + 05***
	(4.19e + 04)	(4.19e + 04)
Shopping centres, sq. m.	4.79e + 03	4.79e + 03
	(2.27e + 04)	(2.27e + 04)
Population density	−5.00e + 04**	−5.00e + 04**
	(2.38e + 04)	(2.38e + 04)
Constant	5.89e + 07	5.89e + 07
	(1.03e + 08)	(1.03e + 08)
Municipality fixed effects	Yes	Yes
Time fixed effects	Yes	Yes
*N*	280	280
r2_w	0.770	0.770
r2_b	0.940	0.940
r2_o	0.937	0.937
N groups	28	28

##### Social benefits for researchers and students

For scientists and researchers, one of the main benefits of working within a research infrastructure, either for applied or fundamental research, is the opportunity to access new experimental data, to contribute to the creation of new knowledge, and, ultimately, to publish scientific papers in scholarly journals. Thus, the unit benefit is the marginal social value of scientific publications. Following this method, we need information about total wage of researchers in Tecnocampus (5,557,174 euros) and the time devoted to research (26,4%).^8^ According to these data, the value of the research at Tecnocampus is 1,467,094 euros.^9^

A second benefit is the reduction of over-education. Some of the Tecnocampus employees obtain benefit from a job that corresponds to their skills and educational level, thanks to Tecnocampus activity. Tecnocampus employs 484 full-time employees (adding researchers, staff, and people from the start-ups) with university studies. This figure increases to 729 workers if we consider indirect and induced impact. Although there is no official statistics on over-education in Spain, Martínez García (2013) calculates this over-education among the active population with university studies, and depending on the definition used, this figure ranges from 25.7 to 38.4%. We use 30.96%, which is within this range and which is obtained using data from the subsample of the Continuous Sample of Labour Force. To evaluate this improvement economically, it is necessary to multiply the number of workers (225,64) for which Tecnocampus has deleted over-education for a price. The price is the sum of two values: first, the amount of money from the training costs for a university student, which have now been used correctly by the society (23,494 euros). In addition, it is necessary to add the opportunity cost. According to the salary structure survey (INE), the average salary of a non-graduate worker is 21,267 euros per year, which, for 4 years, amounts to 85,068 euros. Therefore, the cost of overdraft per person is 108,562 euros. If we multiply this by the number of workers who are no longer over-educated for their work, thanks to the existence of Tecnocampus, we obtain the social benefit for the reduction of over-education: 25,696,443 euros. It is also important to notice that this estimation may be undermining the total social value of reducing over-education in terms of job satisfaction or the increase in performance due to better organisation support (Addimando, 2019).

A third benefit is the graduates’ benefit, which is calculated by the reduction of the future rate of unemployment and by a higher future salary. However, this benefit cannot be attributed to all the students of Tecnocampus, but only to those who would not have continued their studies without Tecnocampus. These students can be of two types: those who study a university degree that does not exist in the rest of the Catalan university offer or those who study because the university is close to their place of residence and have other obligations throughout the week (labour, relatives, etc.). In the first case, Tecnocampus offers a degree that is not offered in any other Catalan university: the degree of Logistics and Maritime Business. It is reasonable to think, especially taking into account the profile of students (many of which have come from higher school specialisation in this subject), who would not have undertaken university studies in the absence of this offer. In the second case, in order to limit these individuals to the maximum, we limit the amount to those Mataró students who declare that they work half-day. In both cases, we only use the fraction of students who considered Tecnocampus as their first option. We will also include students who enjoy a Tecnocampus social scholarship (18 students). In total, we have 160 students. The social return of 1 year of university studies is approximately 7.15% (de La Fuente and Jimeno, 2011). Therefore, all these 160 students will see how, upon finishing their undergraduate studies, their average annual salary as graduated student (35,494 euros) will grow by 7.15%, that is, 2,537.80 euros, thus the total graduates’ benefit of 406,051.36 euros.^10^

Finally, the last benefit is the consumer surplus. It is possible to assume that a part of the students that study in the Tecnocampus would be prepared to pay a greater amount for enrollment. These students would value aspects, such as the innovative offer of some degrees, its focus, or the fact of studying in an STP. This greater value is due to the fact that these features can bring students higher expectations in their future salaries. This benefit has been applied only to the students who choose the Tecnocampus in first preference for a degree with a very restrictive or non-existent offer and the students in first preference, who are from Mataró. These students are 133 and 162, respectively. Properly measuring this consumer surplus implies carrying out a survey exercise on a representative sample of local students asking for their availability to pay above the price actually paid. In this case, we do not have this data. Falconieri et al. (2004) and Kesenne (2005) show that under certain reasonable assumptions regarding the demand curve and price setting, consumer surplus can be approximated by 50% of the enrollment expenses of these local students (which they are in an amount slightly more than 5,000 euros in most cases). The amount of the consumer surplus is 735,500 euros.

##### Social benefits for the region

Although Tecnocampus provides some benefits for Mataró city as a destination, the benefits for Catalonia are negligible. In this section, some quantitative data of the effect of new firm localisation in the territory are presented. Next, we support our analysis with some qualitative data to bring an idea of the benefits for Mataró and Maresme region.

In the model M1 of Table 5, we can observe a significant and positive effect of the lagged dependent variable showing a persistence of localisation effects, which suggests that new companies follow other companies as an evidence of the existence of localisation economies. Public spending at the municipality has a negative, significant, and low-magnitude effect at Maresme, a result that suggests that public spending possibly supports the consolidation of existing companies. The effect of population on firm births is insignificant, although this result contradicts other studies carried out for Catalonia (Arauzo and Manjón, 2004; Arauzo, 2005), and the main reason is that our study includes other variables that measure more specifically the effects of population in new firms’ birth, such as the number of vehicles or the retails commerce measurement. We can see that commercial vehicles represent the positive effect of urbanisation economies, whereas the more traditional vehicles seem to capture an extent of diseconomies of scale resulting from the increase in population density. The presence of different types of banking institutions in the region has no effect on firm creation. Retail commerce measured as the number of shops seems to capture also the positive effect of urbanisation economies, whereas the square metres of shopping centres measures the negative effect of diseconomies of scale resulting from the increase in density.

**TABLE 5:** Tecnocampus new firms localisation effects.

	**M (1)**	**M (2)**
	
	**Base (new firms)**	**TCM effect (new firms)**
	**Coef. (s.d.)**	**Coef. (s.d.)**
Dependent variable (*t*–1)	0.23691***	0.23691***
	(0.06725)	(0.06725)
TCM effect		7.01241**
		(3.14640)
Local public expenditure	−0.00000***	−0.00000***
	(0.00000)	(0.00000)
Population	0.00010	0.00010
	(0.00089)	(0.00089)
Unemployment rate	−0.69734	−0.69734
	(0.53190)	(0.53190)
Telephones	0.00142	0.00142
	(0.00098)	(0.00098)
Automobiles	−0.00538***	−0.00538***
	(0.00205)	(0.00205)
Trucks and cargo vehicles	0.02371***	0.02371***
	(0.00860)	(0.00860)
Other vehicles	−0.00560***	−0.00560***
	(0.00117)	(0.00117)
Banks	−0.40692	−0.40692
	(0.68712)	(0.68712)
Savings banks	−0.22582	−0.22582
	(0.44758)	(0.44758)
Credit unions	6.18973	6.18973
	(4.61280)	(4.61280)
Retail commerce	0.02734***	0.02734***
	(0.00773)	(0.00773)
Shopping centres, sq. m.	−0.00818**	−0.00818**
	(0.00402)	(0.00402)
Population density	−0.00525	−0.00525
	(0.00416)	(0.00416)
Constant	50.69598***	50.69598***
	(18.36093)	(18.36093)
Municipality fixed effects	Yes	Yes
Time fixed effects	Yes	Yes
*N*	280	280
r2_w	0.856	0.856
r2_b	0.835	0.835
r2_o	0.642	0.642
N groups	28	28

*p < 0.1, **p < 0.05, ***p < 0.01.

As part of the effect formerly calculated in *Social Benefits for Companies*, Model M2 of Table 5 adds Tecnocampus’ effect on the territory. The first thing to note is that adding this variable does not change the location results commented before; hence, our controls are effective to capture other local characteristics. It should be noted that the effect is positive and significant at the 5% level in the Maresme region, which confirms the positive social value for the territory of this institution. The result shows that the presence of Tecnocampus has created approximately seven new companies during the period analysed over expectation of the local characteristics. Noticing that the average sales of the companies in our sample during the period of analysis is 603,386 euros, the total annual impact of Tecnocampus in the region can be valued at 4,223,702 euros, considering only the new firm creation. This extra opportunity for firm creation also has some desirable characteristics not measured in our analysis such as the more innovative nature of new firms near entrepreneurial education hubs (Wei et al., 2019).

**FIGURE 1:**
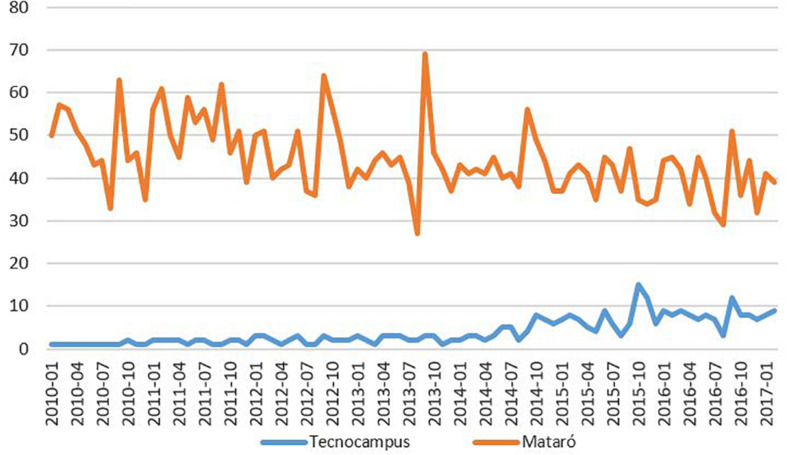
Relevance of Tecnocampus in Mataró.

Second, we have used Google Trends to make an approximation to the destination effect. Figure 1 (see Supplementary Material for quantitative data) shows the evolution of the recognition of the term Tecnocampus in the field “jobs and education” related to the term Mataró during the period 2010–2017. It can be seen that, while Mataró’s visibility in this area has been reduced (values around 50 to values around 40 out of 100), the visibility of Tecnocampus has maintained a clearly increasing evolution from 0 to 10. Furthermore, using a search algorithm of the R programme, we have collected tweets using either the word “Tecnocampus” or the hashtag #Tecnocampus. Throughout the period 2010–2016, Tecnocampus has been mentioned 17,991 times. Likewise, while the hashtag #Mataró has been used 140,672 times the hashtag #Tecnocampus has been used only 3,644 times. More interesting are the interactions between Mataró and Tecnocampus. For example, throughout the period, 4,611 times Mataró and Tecnocampus have been mentioned in the same tweet. These mentions have grown more than 100% from 2010 (307) to 2016 (672). The hashtag #Mataró has been used 1,873 times when the Tecnocampus has been mentioned. The growth in this case has been spectacular: from 22 times in 2010 to 280 times in 2016. When the hashtag #Tecnocampus has been used, Mataró has been mentioned 735 times. In this case, the growth between 2010 and 2016 has been more modest (from 53 to 66 times). Finally, 1,668 times the account @TecnoCampus has been mentioned as well as Mataró. This figure has evolved 22 times in 2010 to 314 times in 2016. Again, growth is very high. We can conclude that the impact of Tecnocampus on social networks has also been growing throughout the period as well as its presence linked to that of the city of Mataró.

Other benefits that have not been considered (and are of small consideration) are in terms of social capital, intangible value, benefits from visitors, and other externalities.

#### Cost

The part of costs included the amount of operating and investment expenses of the Tecnocampus used in the part of economic impact (16,078,921 euros). Additionally, we have to add the cost of enrolment of local students (13,304,074). Therefore, costs amount to 29,082,995 euros.

#### Cost–Benefit Ratio

To sum up, Table 6 shows the results obtained from the analysis. As benefits (69,528,592 euros) outweigh total costs (29,082,995 euros), it can be concluded that the net social benefit from Tecnocampus activity is positive and high (40,445,597 euros). This is a cost–benefit ratio of 2.39, meaning that every euro invested in Tecnocampus activity generates 2.39 euros of social value for their stakeholders, namely, the population of Catalonia. This is high social profitability for the area and its residents.

**TABLE 6:** Social value: summary of benefits and costs (euros).

**Benefits**	**In euros**	**Costs**	**In euros**
***Net revenues***	***13,762,835***	Operating expenses and investment	16,078,921
***Social benefit (SB)***	***55,765,757***	Local students	13,304,074
(1) SB for companies	27,460,669		
*(I) Start-up creation*	*3,719,237*		
*(II) New products*	*3,241,432*		
*(III) Location*	*20,500,000*		
(2) SB for researchers and students	28,305,088		
*(I) Research*	*1,467,094*		
*(II) Over-education*	*25,696,443*		
*(III) Benefits for graduates*	*406,051*		
*(IV) Consumer surplus*	*735,500*		
(3) SB for the region*			
**Total**	**69,528,592**	**Total**	**29,082,995**
**Cost–benefit ratio**	**2.39**		

*The amount that has to do with the formation of seven new enterprises in Maresme region (4,223,702 euros) is already included as part of the social benefits for companies due to location (20,500,000 euros). For this reason, the 4,223,702 euros are not included in this table so as to avoid double accounting.

## Conclusion

In this article, the economic and social impact of an STP on the region has been analysed: Tecnocampus Mataró-Maresme. Rather than adopting a point of view solely based on the traditional impact study carried out on the basis of the effect of investment on local economy, in this work, additionally, a study on the CBA has been incorporated. As this kind of analyses requires more information, they are less frequently used, but they are more appropriate for a detailed and precise analysis in which aspects with an indisputable social value, such as research, start-up creation, or over-occupancy reduction, are monitored. In this study, the different elements composing benefits and costs have been described. Both benefits and costs include social components. Therefore, this article can be regarded as a pioneer, considering that it carries out a social valuation of a public infrastructure that combines research, university education, and entrepreneurship.

The current lack of a standardised and generally accepted procedure for the calculation of the investment’s social profitability (basic indicator of the CBA) in STP requires an eclectic approach in the sense of using a mixed methodology using what has been done in other areas, which are more developed in the analysis of scientific infrastructures [for instance, the infrastructure light Synchrotron (Raya and García-Montalvo, 2016) or super-computers] and the specialties of STPs. Either way, in the study, there are clearly identified and justified the assumptions that have been used.

Hence, the benefits obtained are approximately 2.4 times the incurred costs. Stewart et al. (2019) state that in view of the increase of the pressure upon the public budgets, it is necessary to justify “what do we get for this money” invested in cyberinfrastructures. The aforementioned results clearly justify the profitability of the investment in Tecnocampus. This rate is sufficiently high to resist any sensibleness analysis, particularly if we take into account that the majority of the assumptions adopted in this article have been conservative.

All things considered, economic value represents more than 0.054% of the Catalan GDP, whereas the employment impact represents almost 0.37% of total unemployment in the region. Finally, the total multiplier generated by Tecnocampus activity is 1.89, in line with those previously found in the literature, which range from 1.51 to 2.03 (Moretti and Thulin, 2013; Hermannsson et al., 2014; Zhang et al., 2017). As far as social value is concerned, we conclude that Tecnocampus has a positive and significant impact both on new firm creation and on the productivity effect in profits in Maresme region.

The policy implications of this article are threefold. First, they provide methodology to test the impact of STPs as a heterogeneous and difficult to measure phenomenon. It combines several direct, indirect and induced economic effects, as well as other social effects that should be accounted for to determine the actual value of these initiatives by local governments. Second, the results of this analysis show a positive and significant effect of the presence of an STP in a region in terms of economic and social value. The existence of an external social value represented by spillover benefits for the actual firms is particularly important, as well as a pull effect for new firms within the region, education, and communication effects that favour the region. Finally, the case of Tecnocampus can be seen as success history for other regions that want to incentivise changes in their industrial composition toward a technological and more service-oriented economy.

Future lines of research should focus on capture social value externalities of Tecnocampus and STPs in general at the industry, firm, and individual levels. The strategy in this article has followed a more generalist approach to try to capture all the benefits for specific agents (enterprises, researchers, students, and entrepreneurs) and at the aggregated level (Catalonia, Maresme, and Mataró). The effect of STPs can be decomposed by the specific industrial sectors so that we can gain a better understanding of the main beneficiaries of this type of institutions and understand if crowding-out effects exist. Additionally, we can measure directly the spillover effects at the firm level in terms of productivity and employment generated. If a positive and significant effect of an STP is presented in average in all firms, then the financing of these parks by the private sector can be more easily promoted. Also a natural experiment can be run identifying all the new enterprises in a period of time. The idea will be distinguishing between new firms inside and outside the STP and also identifying firms that have applied and not obtained their inclusion in the STP to have full identification of the effects of the STP in the survival and success of these enterprises. Finally, understanding the effect of STP at an individual level can help to understand the effects on extra years of education, higher incomes, and more stable job offerings. One example can be analysing the fostering of entrepreneurship spirit among students. The idea is measuring the difference with more traditional universities in terms of creating an enterprise after graduation explained in the base of different and more applied curriculum and internship opportunities during the undergraduate years. Also the effect of the STP in the productivity of academic researchers is interesting as more articles can be produced by the easiest access to real information and the more straightforward application of the produced research.

All in all, the main strength of this article is that a combination of different methodologies has been used in order to obtain the social value of an STP. On the other hand, the lack of a standardised methodology for doing so seems to be its main weakness. For this reason, it has been a must to analyse and meditate on the validity of every single attempt made to monetise the social value creation of Tecnocampus. As a matter of fact, another weakness of the article is the difficulty in obtaining appropriate data in order to use it in the framework of the proposed methodology. All things considered, future research activities – along with the future lines of research suggested in the former paragraph – should address these limitations, particularly in regard to the creation of a standardised methodology to assess social value of STP. Developing this standardised methodology, which will include the data necessary to conduct it, would facilitate the successive measurement of social value creation of STPs, allowing the creation of key performance indicators to manage this kind of institution under a proper multistakeholders’ approach.

## Data Availability Statement

The raw data supporting the conclusions of this article will be made available by the authors, without undue reservation.

## Ethics Statement

Ethical review and approval was not required for the study on human participants in accordance with the local legislation and institutional requirements. Written informed consent for participation was not required for this study in accordance with the national legislation and the institutional requirements.

## Author Contributions

All authors listed have made a substantial, direct and intellectual contribution to the work, and approved it for publication.

## Conflict of Interest

The authors are employees of Fundació Tecnocampus Mataró-Maresme.

## Footnotes

1 SABI is the acronym for Iberian Balance Analysis System. It is a private database that reflects the status of more than one million companies in Spain and Portugal done by Bureau Van Dijk.

2 Maresme region is formed by 30 municipalities, two of which are excluded (Òrrius and Sant Iscle de Vallalta) because they have fewer than 1,000 inhabitants. So, not all the relevant variables for the analysis can be obtained.

3 Following Jeong et al. (2015), mischievous practices in economic impact analyses are avoided. These mischievous practices are related to population considered and technical issues (apart of not considering increase in monetary and non-monetary costs and benefits than can also be generated by Tecnocampus’ activity).

4 Catalan GDP in 2016 was 223,629 million euros and total unemployment at the end of 2016 was above 497,400 (according to the Idescat, the Catalan Statistical Office).

5 As in the case of the economic impact, we have calculated CBA for Mataró city, which is available by request.

6 In fact, this negative effect is not clear because people who come to Tecnocampus tend to use less congested routes, in particular those coming from Barcelona.

7 A higher rate. In Catalonia, this rate was of 1 every 535.

8 33% according to “The use of the time by the Spanish university lecturers” (Ministry of Health Social Services and Equality, 2016). In the case of Tecnocampus, given the characteristics of the centre (greater time devoted to teaching and management tasks by lecturers) it is reasonable to think that this percentage is lower. On average, we will consider that it is 20% lower (26.4%).

9 Despite the consistency with the principles of the CBA of evaluating the impact of research on the marginal cost of production, it can be considered that this method undervalues research because it does not incorporate the social return that has the public financing of research.

10 This figure can be higher if we consider that Tecnocampus offer some extra programmes for graduates, especially in entrepreneurship.
